# Supplementary figures and images for: Associations between pre-stroke physical activity and physical quality of life three months after stroke in patients with mild disability
Source: PLoS One. 2022 Jun 29;17(6):e0266318. doi: 10.1371/journal.pone.0266318 (PMC9242505; doi:10.1371/journal.pone.0266318)

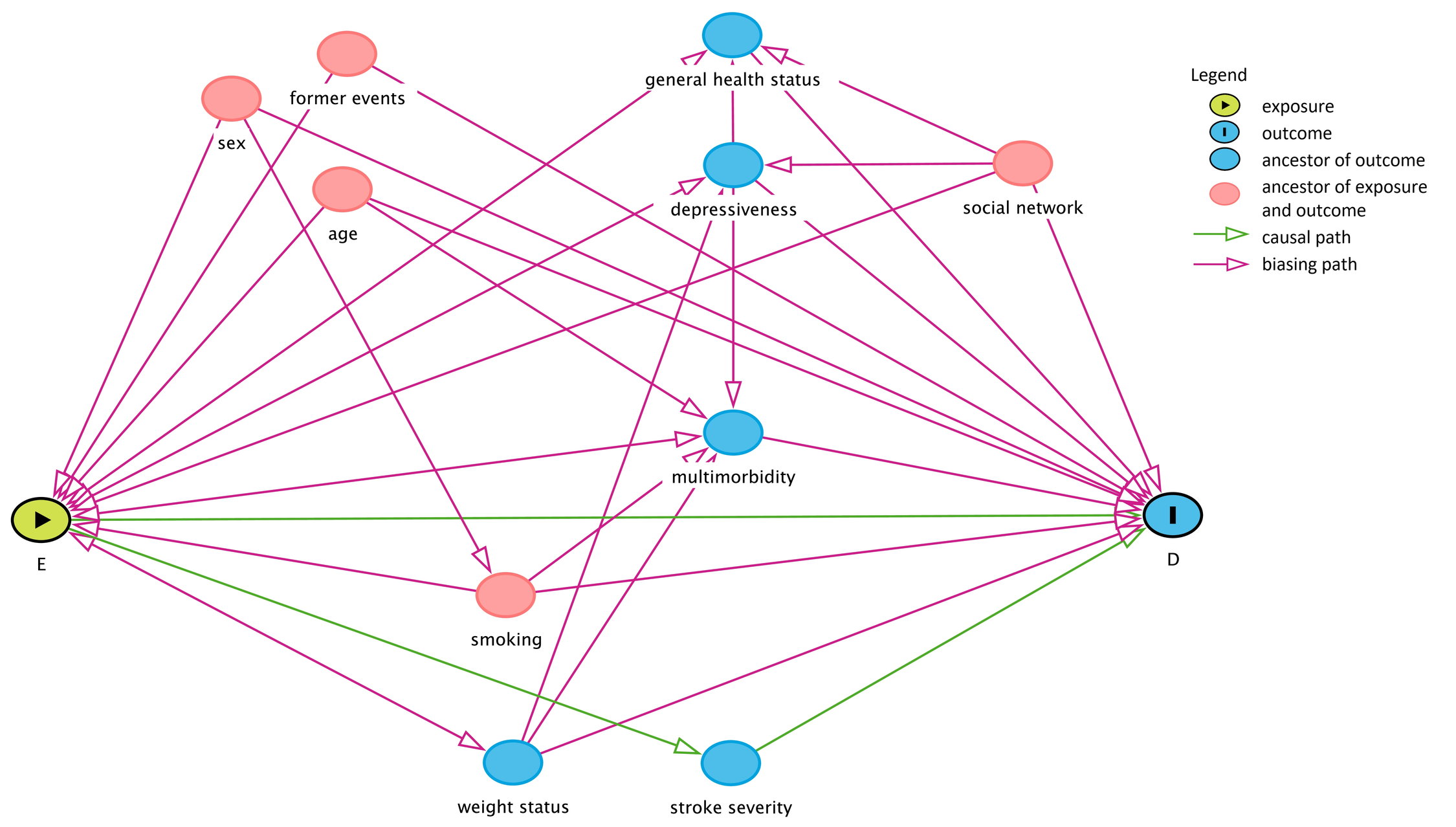

Supplement: S1 Fig — (TIF) [file pone.0266318.s001.tif]

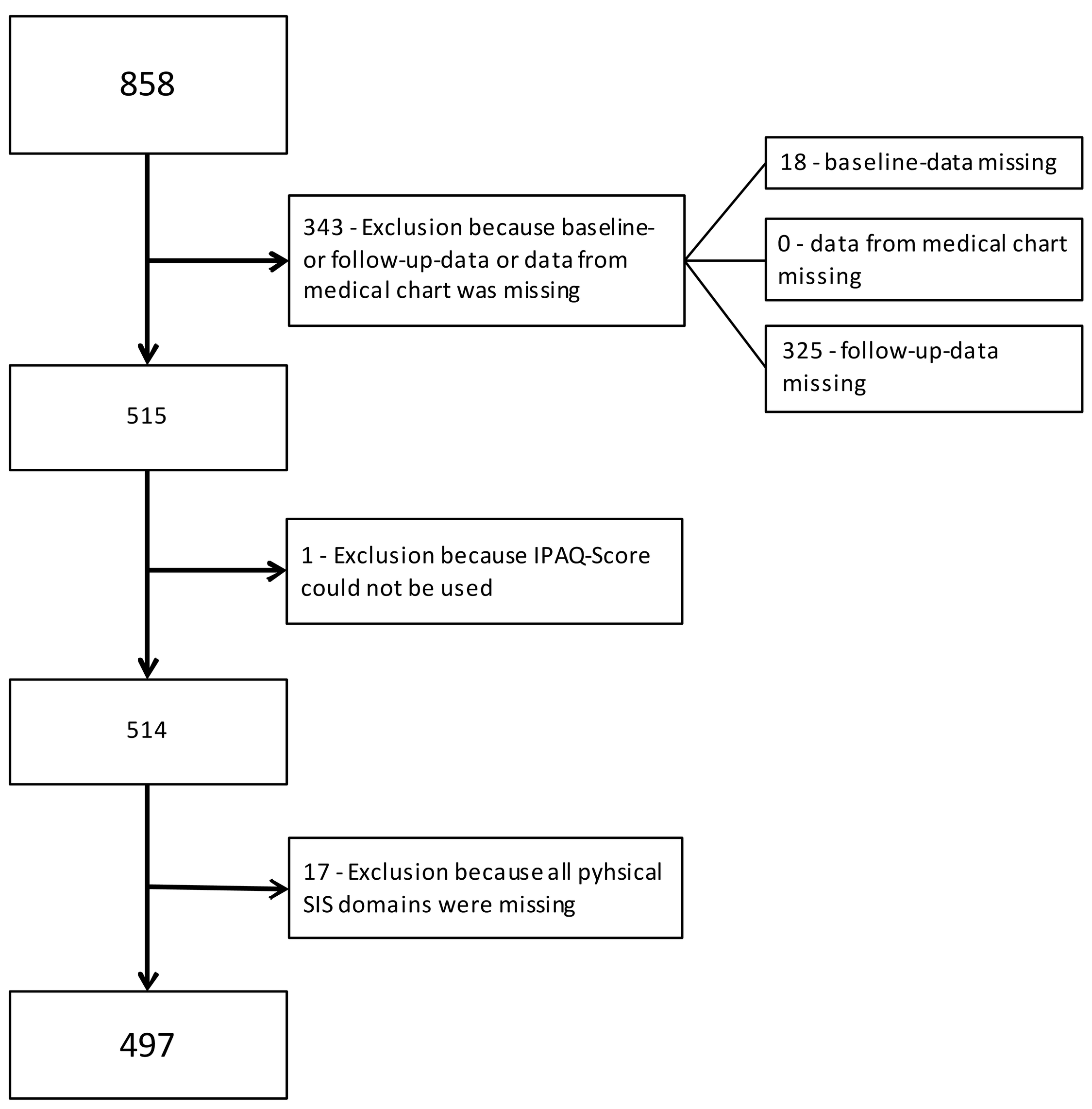

Supplement: S2 Fig — (TIF) [file pone.0266318.s002.tif]

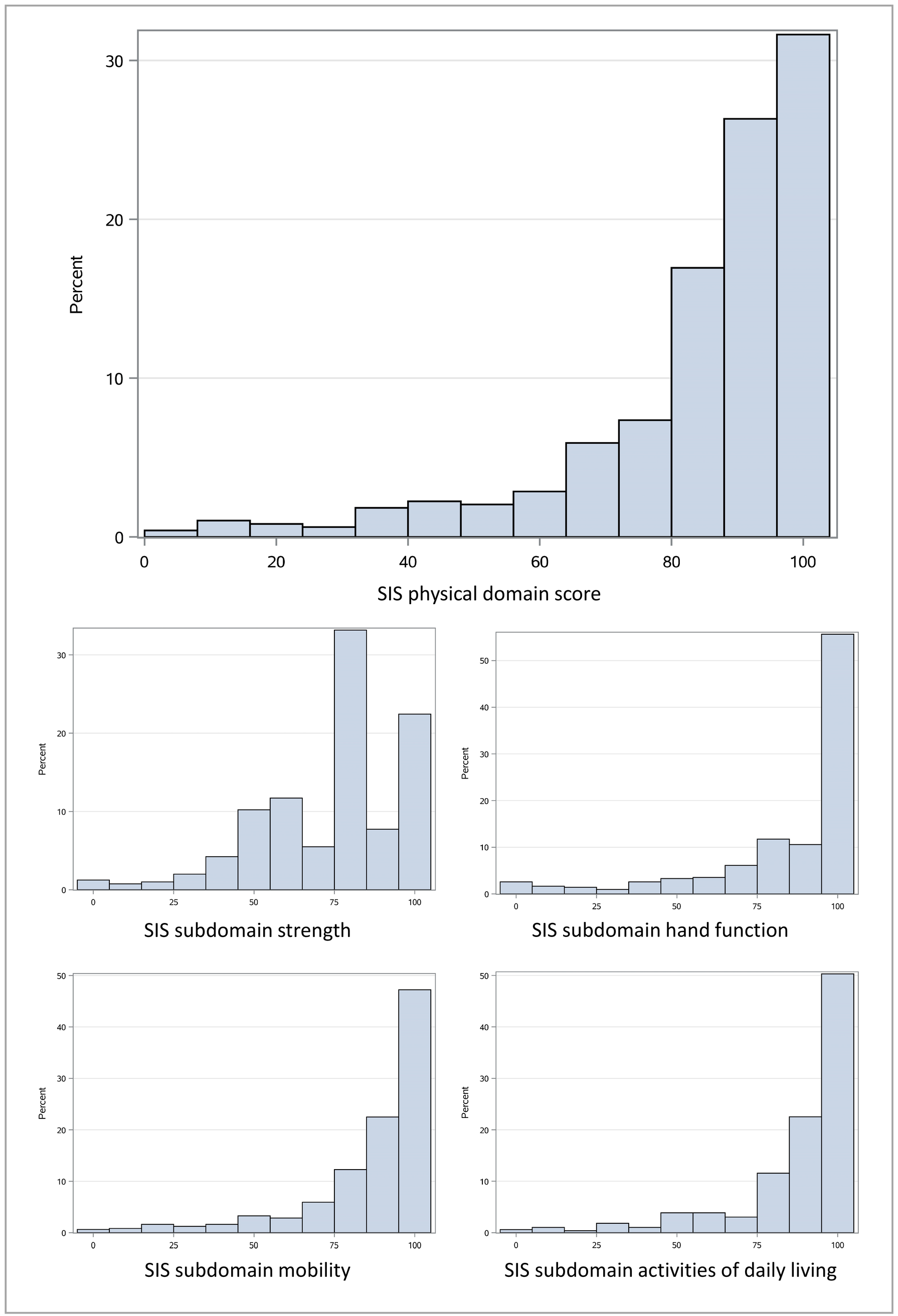

Supplement: S3 Fig — (TIF) [file pone.0266318.s003.tif]
