# Supplementary material for: Associations between pre-stroke physical activity and physical quality of life three months after stroke in patients with mild disability
Source: PLoS One. 2022 Jun 29;17(6):e0266318. doi: 10.1371/journal.pone.0266318 (PMC9242505; doi:10.1371/journal.pone.0266318)
Supplement: S1 Table — (DOCX) [file pone.0266318.s004.docx]

References

1. Jurevičienė E, Onder G, Visockienė Ž, Puronaitė R, Petrikonytė D, Gargalskaitė U, et al. Does multimorbidity still remain a matter of the elderly: Lithuanian national data analysis. Health Policy. 2018; 122:681–6. doi: 10.1016/j.healthpol.2018.03.003 PMID: 29602577.

2. Sakib MN, Shooshtari S, St John P, Menec V. The prevalence of multimorbidity and associations with lifestyle factors among middle-aged Canadians: an analysis of Canadian Longitudinal Study on Aging data. BMC Public Health. 2019; 19:243. doi: 10.1186/s12889-019-6567-x PMID: 30819126.

3. Varma VR, Dey D, Leroux A, Di J, Urbanek J, Xiao L, et al. Re-evaluating the effect of age on physical activity over the lifespan. Preventive Medicine. 2017; 101:102–8. doi: 10.1016/j.ypmed.2017.05.030 PMID: 28579498.

4. Twardzik E, Clarke P, Elliott MR, Haley WE, Judd S, Colabianchi N. Neighborhood Socioeconomic Status and Trajectories of Physical Health-Related Quality of Life Among Stroke Survivors. Stroke. 2019; 50:3191–7. doi: 10.1161/STROKEAHA.119.025874 PMID: 31526122.

5. Field MJ, Gebruers N, Shanmuga Sundaram T, Nicholson S, Mead G. Physical Activity after Stroke: A Systematic Review and Meta-Analysis. ISRN Stroke. 2013; 2013:1–13. doi: 10.1155/2013/464176.

6. Béjot Y, Bailly H, Durier J, Giroud M. Epidemiology of stroke in Europe and trends for the 21st century. Presse Med. 2016; 45:e391-e398. doi: 10.1016/j.lpm.2016.10.003 PMID: 27816343.

7. Zhu W, Jiang Y. Determinants of quality of life in patients with hemorrhagic stroke: A path analysis. Medicine (Baltimore). 2019; 98:e13928. doi: 10.1097/MD.0000000000013928 PMID: 30702554.

8. Prisnie JC, Sajobi TT, Wang M, Patten SB, Fiest KM, Bulloch AGM, et al. Effects of depression and anxiety on quality of life in five common neurological disorders. Gen Hosp Psychiatry. 2018; 52:58–63. doi: 10.1016/j.genhosppsych.2018.03.009 PMID: 29684713.

9. Robinson RG, Jorge RE. Post-Stroke Depression: A Review. Am J Psychiatry. 2016; 173:221–31. doi: 10.1176/appi.ajp.2015.15030363 PMID: 26684921.

10. Patel MD, McKevitt C, Lawrence E, Rudd AG, Wolfe CDA. Clinical determinants of long-term quality of life after stroke. Age Ageing. 2007; 36:316–22. doi: 10.1093/ageing/afm014 PMID: 17374601.

11. Mikkelsen K, Stojanovska L, Polenakovic M, Bosevski M, Apostolopoulos V. Exercise and mental health. Maturitas. 2017; 106:48–56. doi: 10.1016/j.maturitas.2017.09.003 PMID: 29150166.

12. Ohrnberger J, Fichera E, Sutton M. The relationship between physical and mental health: A mediation analysis. Soc Sci Med. 2017; 195:42–9. doi: 10.1016/j.socscimed.2017.11.008 PMID: 29132081.

13. Gallacher KI, Jani BD, Hanlon P, Nicholl BI, Mair FS. Multimorbidity in Stroke. Stroke. 2019; 50:1919–26. doi: 10.1161/STROKEAHA.118.020376 PMID: 31233391.

14. Anokye NK, Trueman P, Green C, Pavey TG, Taylor RS. Physical activity and health related quality of life. BMC Public Health. 2012; 12:624. doi: 10.1186/1471-2458-12-624 PMID: 22871153.

15. Granger E, Di Nardo F, Harrison A, Patterson L, Holmes R, Verma A. A systematic review of the relationship of physical activity and health status in adolescents. Eur J Public Health. 2017; 27:100–6. doi: 10.1093/eurpub/ckw187 PMID: 28340201.

16. Jakicic JM. The effect of physical activity on body weight. Obesity (Silver Spring). 2009; 17 Suppl 3:S34-8. doi: 10.1038/oby.2009.386 PMID: 19927144.

17. Chin S-H, Kahathuduwa CN, Binks M. Physical activity and obesity: what we know and what we need to know. Obes Rev. 2016; 17:1226–44. doi: 10.1111/obr.12460 PMID: 27743411.

18. Jezewska-Zychowicz M, Gębski J, Plichta M, Guzek D, Kosicka-Gębska M. Diet-Related Factors, Physical Activity, and Weight Status in Polish Adults. Nutrients. 2019; 11. doi: 10.3390/nu11102532 PMID: 31640114.

19. Chekroud SR, Gueorguieva R, Zheutlin AB, Paulus M, Krumholz HM, Krystal JH, et al. Association between physical exercise and mental health in 1·2 million individuals in the USA between 2011 and 2015: a cross-sectional study. The Lancet Psychiatry. 2018; 5:739–46. doi: 10.1016/S2215-0366(18)30227-X.

20. Craft LL, Perna FM. The Benefits of Exercise for the Clinically Depressed. Prim Care Companion J Clin Psychiatry. 2004; 6:104–11. doi: 10.4088/pcc.v06n0301 PMID: 15361924.

21. Wendel-Vos GCW, Schuit AJ, Feskens EJM, Boshuizen HC, Verschuren WMM, Saris WHM, et al. Physical activity and stroke. A meta-analysis of observational data. Int J Epidemiol. 2004; 33:787–98. doi: 10.1093/ije/dyh168 PMID: 15166195.

22. Wen C-P, Liu C-H, Jeng J-S, Hsu S-P, Chen C-H, Lien L-M, et al. Pre-stroke physical activity is associated with fewer post-stroke complications, lower mortality and a better long-term outcome. Eur J Neurol. 2017; 24:1525–31. doi: 10.1111/ene.13463 PMID: 28926165.

23. Subramaniam M, Zhang Y, Lau JH, Vaingankar JA, Abdin E, Chong SA, et al. Patterns of physical activity and health-related quality of life amongst patients with multimorbidity in a multi-ethnic Asian population. BMC Public Health. 2019; 19:1612. doi: 10.1186/s12889-019-7941-4 PMID: 31791301.

24. Azevedo MR, Araújo CLP, Reichert FF, Siqueira FV, da Silva MC, Hallal PC. Gender differences in leisure-time physical activity. Int J Public Health. 2007; 52:8–15. doi: 10.1007/s00038-006-5062-1 PMID: 17966815.

25. Seitz N-N, Lochbühler K, Atzendorf J, Rauschert C, Pfeiffer-Gerschel T, Kraus L. Trends In Substance Use And Related Disorders: Analysis of the Epidemiological Survey of Substance Abuse 1995 to 2018. Dtsch Arztebl Int. 2019; 116:585–91. doi: 10.3238/arztebl.2019.0585 PMID: 31587706.

26. Efendi V, Özalevli S, Naz İ, Kılınç O. The effects of smoking on body composition, pulmonary function, physical activity and health-related quality of life among healthy women. Tuberk Toraks. 2018; 66:101–8. doi: 10.5578/tt.50724 PMID: 30246652.

27. Epstein KA, Viscoli CM, Spence JD, Young LH, Inzucchi SE, Gorman M, et al. Smoking cessation and outcome after ischemic stroke or TIA. Neurology. 2017; 89:1723–9. doi: 10.1212/WNL.0000000000004524 PMID: 28887378.

28. Fortin M, Haggerty J, Almirall J, Bouhali T, Sasseville M, Lemieux M. Lifestyle factors and multimorbidity: a cross sectional study. BMC Public Health. 2014; 14:686. doi: 10.1186/1471-2458-14-686 PMID: 24996220.

29. Béjot Y, Jacquin A, Daubail B, Lainay C, Janoura S, Aboa-Eboulé C, et al. Smoking status and severity of ischemic stroke. A population-based study. Eur Neurol. 2014; 71:59–64. doi: 10.1159/000355021 PMID: 24334964.

30. Lindsay Smith G, Banting L, Eime R, O'Sullivan G, van Uffelen JGZ. The association between social support and physical activity in older adults: a systematic review. Int J Behav Nutr Phys Act. 2017; 14:1–21. doi: 10.1186/s12966-017-0509-8 PMID: 28449673.

31. Hunter RF, Davis M, Tully MA, Kee F. Physical activity buddies: a network analysis of social aspects of physical activity in adults. The Lancet. 2012; 380:S51. doi: 10.1016/S0140-6736(13)60407-9.

32. Moschny A, Platen P, Klaassen-Mielke R, Trampisch U, Hinrichs T. Physical activity patterns in older men and women in Germany: a cross-sectional study. BMC Public Health. 2011; 11:559. doi: 10.1186/1471-2458-11-559 PMID: 21752288.

33. Bergland A, Meaas I, Debesay J, Brovold T, Jacobsen EL, Antypas K, et al. Associations of social networks with quality of life, health and physical functioning. European Journal of Physiotherapy. 2016; 18:78–88. doi: 10.3109/21679169.2015.1115554.

34. Kawachi I, Berkman LF. Social ties and mental health. J Urban Health. 2001; 78:458–67. doi: 10.1093/jurban/78.3.458 PMID: 11564849.

35. Park NS, Jang Y, Lee BS, Chiriboga DA, Chang S, Kim SY. Associations of a social network typology with physical and mental health risks among older adults in South Korea. Aging Ment Health. 2018; 22:631–8. doi: 10.1080/13607863.2017.1286456 PMID: 28290722.

36. Ramos-Lima MJM, Brasileiro IdC, Lima TL de, Braga-Neto P. Quality of life after stroke: impact of clinical and sociodemographic factors. Clinics (Sao Paulo). 2018; 73:e418. doi: 10.6061/clinics/2017/e418 PMID: 30304300.

37. Laxy M, Holle R, Döring A, Peters A, Hunger M. The longitudinal association between weight change and health-related quality of life: the KORA S4/F4 cohort study. Int J Public Health. 2014; 59:279–88. doi: 10.1007/s00038-013-0506-x PMID: 23999627.

38. López-García E, Banegas Banegas JR, Gutiérrez-Fisac JL, Pérez-Regadera AG, Gañán LD, Rodríguez-Artalejo F. Relation between body weight and health-related quality of life among the elderly in Spain. Int J Obes Relat Metab Disord. 2003; 27:701–9. doi: 10.1038/sj.ijo.0802275 PMID: 12833114.

39. Avila C, Holloway AC, Hahn MK, Morrison KM, Restivo M, Anglin R, et al. An Overview of Links Between Obesity and Mental Health. Curr Obes Rep. 2015; 4:303–10. doi: 10.1007/s13679-015-0164-9 PMID: 26627487.
