# Supplementary material for: Associations between pre-stroke physical activity and physical quality of life three months after stroke in patients with mild disability
Source: PLoS One. 2022 Jun 29;17(6):e0266318. doi: 10.1371/journal.pone.0266318 (PMC9242505; doi:10.1371/journal.pone.0266318)
Supplement: S2 Table — (DOCX) [file pone.0266318.s005.docx]

| **S2 Table. Differences of included and excluded patients** | | | | | | | |  |
| --- | --- | --- | --- | --- | --- | --- | --- | --- |
|  | Included | | | Excluded | | | test statistic | p-value |
|  | 497 (57.9)^1^ | | | 361 (42.1) | | |  |  |
| Variable | n | Value | | n | Value | |  |  |
| Age in years*, mean (SD^2^)* | 481 | 69,6 | (12,5) | 226 | 69,6 | (13,9) | 0,3 | 0.751^a^ |
| Depressiveness: PHQ-Score*, mean (SD)* | 467 | 4,8 | (4,3) | 208 | 5,7 | (4,8) | 2,2 | 0.0279^a^ |
| General health, *mean (SD)* | 480 | 3,1 | (0,9) | 224 | 3,4 | (0,9) | 4,2 | <.0001^a^ |
| Stroke Severity |  |  |  |  |  |  |  |  |
| mRS^4^*, median (IR^5^)* | 488 | 2,0 | (1,0) | 360 | 3,0 | (2,0) | 2,7 | 0.0073^a^ |
| NIHSS^6^*, mean (SD)* | 487 | 2,8 | (3,9) | 360 | 3,8 | (5,0) | 2,4 | 0.0147^a^ |
| Sex |  |  |  |  |  |  |  |  |
| Male |  | 283 | (58,8) |  | 126 | (55,8) | 0,6 | 0.4387^b^ |
| Female |  | 198 | (41,2) |  | 100 | (44,3) |  |  |
| Physical activity |  |  |  |  |  |  |  |  |
| Low |  | 273 | (54,9) |  | 243 | (70,9) | 24,8 | <.0001^b^ |
| Moderate |  | 94 | (18,9) |  | 31 | (9,0) |  |  |
| High |  | 130 | (26,2) |  | 69 | (20,1) |  |  |
| Social network |  |  |  |  |  |  |  |  |
| Solitarily |  | 123 | (26,1) |  | 137 | (63,7) | 7,5 | 0.0063^b^ |
| Cohabiting |  | 349 | (73,9) |  | 78 | (36,3) |  |  |
| Weight status |  |  |  |  |  |  |  |  |
| BMI^7^ < 30 |  | 363 | (74,1) |  | 281 | (78,9) | 2,7 | 0.1023^b^ |
| BMI ≥ 30 |  | 127 | (25,9) |  | 75 | (21,1) |  |  |
| Smoking |  |  |  |  |  |  |  |  |
| Current |  | 71 | (14,8) |  | 43 | (19,3) | 2,3 | 0.3234^b^ |
| Former |  | 212 | (44,3) |  | 95 | (42,6) |  |  |
| Never |  | 196 | (40,9) |  | 85 | (38,1) |  |  |
| Former stroke |  |  |  |  |  |  |  |  |
| Yes |  | 124 | (25,1) |  | 93 | (26,1) | 0,1 | 0.7539^b^ |
| No |  | 370 | (74,9) |  | 264 | (74,0) |  |  |
| Multimorbidity |  |  |  |  |  |  |  |  |
| Yes |  | 390 | (78,5) |  | 277 | (76,7) | 0,4 | 0.5454^b^ |
| No |  | 107 | (21,5) |  | 84 | (23,3) |  |  |
| 1 Values are expressed as numbers (percentage) unless otherwise indicated. | | | | | | |  |  |
| 2 Standard deviation |  |  |  |  |  |  |  |  |
|  |  |  |  |  | a Mann-Whitney U Test | | |  |
| 4 modified Rankin Scale |  |  |  |  | b Pearson chi-square | | |  |
| 5 Interquartile range |  |  |  |  |  |  |  |  |
| 6 National Institutes of Health Stroke Scale | | |  |  |  |  |  |  |
| 7 Body Mass Index, BMI = kg/m² |  |  |  |  |  |  |  |  |
