# Supplementary material for: Associations between pre-stroke physical activity and physical quality of life three months after stroke in patients with mild disability
Source: PLoS One. 2022 Jun 29;17(6):e0266318. doi: 10.1371/journal.pone.0266318 (PMC9242505; doi:10.1371/journal.pone.0266318)
Supplement: S3 Table — (DOCX) [file pone.0266318.s006.docx]

| **S3 Table. Results the post-hoc-tests: p-values for the comparison of the three groups**  **of pre-stroke physical activity** | | | | | | | | | | |  |
| --- | --- | --- | --- | --- | --- | --- | --- | --- | --- | --- | --- |
|  | Pre-stroke physical activity | | |  |  |  |  | |  |  | |
| Stroke Impact Scale | Moderate vs. Low | Moderate vs. High | Low vs. High |  |  |  | |  |  |  | |
| Physical domain | 0.0057^1^ | 0,8367 | <.0001 |  |  |  | |  |  |  | |
| Strength | 0,0199 | 0,9842 | 0,0023 |  |  |  | |  |  |  | |
| Hand function | 0,0288 | 0,9675 | 0,0026 |  |  |  | |  |  |  | |
| Mobility | 0,0362 | 0,8010 | 0,0014 |  |  |  | |  |  |  | |
| Activities of daily living | 0,0261 | 0,7540 | 0,0002 |  |  |  | |  |  |  | |
| 1 All p-values were calculated with the Dwass-Steel-Critchlow-Fligner test | | |  |  |  |  |  | |  |  | |
