# Supplementary material for: Associations between pre-stroke physical activity and physical quality of life three months after stroke in patients with mild disability
Source: PLoS One. 2022 Jun 29;17(6):e0266318. doi: 10.1371/journal.pone.0266318 (PMC9242505; doi:10.1371/journal.pone.0266318)
Supplement: S4 Table — (DOCX) [file pone.0266318.s007.docx]

| **S4 Table. Associations between pre-stroke physical activity and SIS strength after three months: Results of the multiple linear regression analysis** | | | |
| --- | --- | --- | --- |
|  |  |  |  |
| Variable | Beta | (95 % CI^1^) | p-value |
| Intercept | 194,1 | (59.8 to 328.5) | 0,0048 |
| Physical activity_high | 4,2 | (-0.7 to 9.1) | 0,0931 |
| Physical activity_moderate | 5,1 | (-0.3 to 10.5) | 0,0633 |
| Physical activity_low | Ref.^2^ |  |  |
| Age | -5,6 | (-12.3 to 1.2) | 0,1041 |
| Age*Age^3^ | 0,1 | (0 to 0.2) | 0,1107 |
| Age*Age*Age^4^ | 0,0 | (0 to 0) | 0,1105 |
| Sex_female | 1,3 | (-3.1 to 5.6) | 0,5580 |
| Sex_male | Ref. |  |  |
| Multimorbidity_no | -0,5 | (-5.8 to 4.8) | 0,8465 |
| Multimorbidity_yes | Ref. |  |  |
| EQVAS^5^ | 1,2 | (-8.8 to 11.3) | 0,8074 |
| EQVAS*EQVAS^6^ | -1,2 | (-2.8 to 0.4) | 0,1553 |
| PHQ^7^ | -0,8 | (-2.1 to 0.4) | 0,1911 |
| PHQ*PHQ^8^ | 0,0 | (-0.1 to 0.1) | 0,4433 |
| BMI^9^ < 30 | 3,9 | (-0.6 to 8.5) | 0,0904 |
| BMI ≥ 30 | Ref. |  |  |
| Social network_cohabiting | 3,6 | (-1.4 to 8.6) | 0,1555 |
| Social network_solitarily | Ref. |  |  |
| Smoking_current | 2,7 | (-3.8 to 9.2) | 0,4138 |
| Smoking_former | -0,8 | (-5.4 to 3.7) | 0,7216 |
| Smoking_never | Ref. |  |  |
| Former stroke_no | 3,4 | (-1.5 to 8.2) | 0,1757 |
| Former stroke_yes | Ref. |  |  |
| NIHSS^10^ | -1,0 | (-1.8 to -0.2) | 0,0165 |
| mRS^11^_2 | 1,0 | (-6 to 8) | 0,7734 |
| mRS_3 | -0,8 | (-7.4 to 5.9) | 0,8256 |
| mRS_4 | -5,2 | (-12.7 to 2.2) | 0,1654 |
| mRS_5 | -2,1 | (-10.2 to 6.1) | 0,6179 |
| mRS_6 | 10,4 | (-7 to 27.7) | 0,2395 |
| mRS_1 | Ref. |  |  |
| 1 Confidence Interval | 9 Body Mass Index, BMI = kg/m² | |  |
| 2 Reference Group | 10 National Institutes of Health Stroke Scale | | |
| 3 Age variable, squared | 11 European Quality of Life visual analogue scale | | |
| 4 Age variable, cubed |  |  |  |
| 5 European Quality of Life visual analogue scale (general health status) | | |  |
| 6 EQVAS variable, squared |  |  |  |
| 7 Patient Health Questionnaire (depressiveness) | |  |  |
| 8 PHQ variable, squared |  |  |  |
