# Supplementary material for: Associations between pre-stroke physical activity and physical quality of life three months after stroke in patients with mild disability
Source: PLoS One. 2022 Jun 29;17(6):e0266318. doi: 10.1371/journal.pone.0266318 (PMC9242505; doi:10.1371/journal.pone.0266318)
Supplement: S5 Table — (DOCX) [file pone.0266318.s008.docx]

| **S5 Table. Associations between pre-stroke physical activity and SIS hand function after three months: Results of the multiple linear regression analysis** | | | |
| --- | --- | --- | --- |
|  |  |  |  |
| Variable | Beta | (95 % CI^1^) | p-value |
| Intercept | 210,1 | (67.2 to 353) | 0,0041 |
| Physical activity_high | 5,6 | (0.6 to 10.7) | 0,0298 |
| Physical activity_moderate | 5,3 | (-0.1 to 10.8) | 0,0565 |
| Physical activity_low | Ref.^2^ |  |  |
| Age | -6,7 | (-13.8 to 0.5) | 0,0674 |
| Age*Age^3^ | 0,1 | (0 to 0.2) | 0,0590 |
| Age*Age*Age^4^ | 0,0 | (0 to 0) | 0,0524 |
| Sex_female | 0,3 | (-4.2 to 4.8) | 0,8943 |
| Sex_male | Ref. |  |  |
| Multimorbidity_no | -1,2 | (-6.6 to 4.2) | 0,6571 |
| Multimorbidity_yes | Ref. |  |  |
| EQVAS^5^ | 6,7 | (-3.5 to 16.9) | 0,1999 |
| EQVAS*EQVAS^6^ | -2,0 | (-3.6 to -0.4) | 0,0167 |
| PHQ^7^ | 0,4 | (-0.9 to 1.7) | 0,5337 |
| PHQ*PHQ^8^ | -0,1 | (-0.2 to 0) | 0,0915 |
| BMI^9^ < 30 | -1,9 | (-6.7 to 2.9) | 0,4282 |
| BMI ≥ 30 | Ref. |  |  |
| Social network_cohabiting | 5,2 | (0 to 10.3) | 0,0508 |
| Social network_solitarily | Ref. |  |  |
| Smoking_current | 4,6 | (-2 to 11.2) | 0,1697 |
| Smoking_former | 2,4 | (-2.3 to 7.1) | 0,3230 |
| Smoking_never | Ref. |  |  |
| Former stroke_no | 4,8 | (-0.3 to 9.8) | 0,0628 |
| Former stroke_yes | Ref. |  |  |
| NIHSS^10^ | -0,9 | (-1.7 to 0) | 0,0382 |
| mRS^11^_2 | 1,6 | (-5.7 to 8.9) | 0,6669 |
| mRS_3 | -1,6 | (-8.6 to 5.3) | 0,6415 |
| mRS_4 | -10,9 | (-18.5 to -3.3) | 0,0049 |
| mRS_5 | -11,2 | (-19.5 to -2.8) | 0,0089 |
| mRS_6 | 4,1 | (-13.9 to 22.2) | 0,6523 |
| mRS_1 | Ref. |  |  |
| 1 Confidence Interval | 9 Body Mass Index, BMI = kg/m² | |  |
| 2 Reference Group | 10 National Institutes of Health Stroke Scale | | |
| 3 Age variable, squared | 11 European Quality of Life visual analogue scale | | |
| 4 Age variable, cubed |  |  |  |
| 5 European Quality of Life visual analogue scale (general health status) | | |  |
| 6 EQVAS variable, squared |  |  |  |
| 7 Patient Health Questionnaire (depressiveness) | |  |  |
| 8 PHQ variable, squared |  |  |  |
