# Supplementary material for: Associations between pre-stroke physical activity and physical quality of life three months after stroke in patients with mild disability
Source: PLoS One. 2022 Jun 29;17(6):e0266318. doi: 10.1371/journal.pone.0266318 (PMC9242505; doi:10.1371/journal.pone.0266318)
Supplement: S6 Table — (DOCX) [file pone.0266318.s009.docx]

| **S6 Table. Associations between pre-stroke physical activity and SIS mobility after three months: Results of the multiple linear regression analysis** | | | |
| --- | --- | --- | --- |
|  |  |  |  |
| Variable | Beta | (95 % CI^1^) | p-value |
| Intercept | 185,9 | (86.8 to 285) | 0,0003 |
| Physical activity_high | 4,1 | (0.7 to 7.6) | 0,0200 |
| Physical activity_moderate | 4,9 | (1.2 to 8.7) | 0,0104 |
| Physical activity_low | Ref.^2^ |  |  |
| Age | -5,8 | (-10.7 to -0.9) | 0,0201 |
| Age*Age^3^ | 0,1 | (0 to 0.2) | 0,0081 |
| Age*Age*Age^4^ | Ref. | (0 to 0) | 0,0026 |
| Sex_female | 2,6 | (-0.5 to 5.7) | 0,1018 |
| Sex_male | 0,0 | (0 to 0) |  |
| Multimorbidity_no | -0,6 | (-4.3 to 3.1) | 0,7451 |
| Multimorbidity_yes | Ref. |  |  |
| EQVAS^5^ | 7,2 | (0 to 14.4) | 0,0509 |
| EQVAS*EQVAS^6^ | -2,0 | (-3.1 to -0.8) | 0,0007 |
| PHQ^7^ | 0,4 | (-0.5 to 1.3) | 0,4199 |
| PHQ*PHQ^8^ | -0,1 | (-0.1 to 0) | 0,0168 |
| BMI^9^ < 30 | 3,3 | (0 to 6.7) | 0,0492 |
| BMI ≥ 30 | Ref. | (0 to 0) |  |
| Social network_cohabiting | 0,6 | (-2.9 to 4) | 0,7393 |
| Social network_solitarily | Ref. |  |  |
| Smoking_current | 2,0 | (-2.7 to 6.7) | 0,4137 |
| Smoking_former | 1,0 | (-2.2 to 4.3) | 0,5299 |
| Smoking_never | Ref. | (0 to 0) |  |
| Former stroke_no | 3,4 | (0 to 6.9) | 0,0525 |
| Former stroke_yes | Ref. |  |  |
| NIHSS^10^ | -0,8 | (-1.4 to -0.2) | 0,0059 |
| mRS^11^_2 | 0,4 | (-4.5 to 5.3) | 0,8754 |
| mRS_3 | -0,8 | (-5.4 to 3.7) | 0,7206 |
| mRS_4 | -4,9 | (-10 to 0.2) | 0,0598 |
| mRS_5 | -8,8 | (-14.6 to -3.1) | 0,0026 |
| mRS_6 | -0,2 | (-13.3 to 12.9) | 0,9755 |
| mRS_1 | Ref. |  |  |
| 1 Confidence Interval | 9 Body Mass Index, BMI = kg/m² | |  |
| 2 Reference Group | 10 National Institutes of Health Stroke Scale | | |
| 3 Age variable, squared | 11 European Quality of Life visual analogue scale | | |
| 4 Age variable, cubed |  |  |  |
| 5 European Quality of Life visual analogue scale (general health status) | | |  |
| 6 EQVAS variable, squared |  |  |  |
| 7 Patient Health Questionnaire (depressiveness) | |  |  |
| 8 PHQ variable, squared |  |  |  |
