# Supplementary material for: Associations between pre-stroke physical activity and physical quality of life three months after stroke in patients with mild disability
Source: PLoS One. 2022 Jun 29;17(6):e0266318. doi: 10.1371/journal.pone.0266318 (PMC9242505; doi:10.1371/journal.pone.0266318)
Supplement: S7 Table — (DOCX) [file pone.0266318.s010.docx]

| **S7 Table. Associations between pre-stroke physical activity and SIS activities of daily living after three months: Results of the multiple linear regression analysis** | | | |
| --- | --- | --- | --- |
|  |  |  |  |
| Variable | Beta | (95 % CI^1^) | p-value |
| Intercept | 193,6 | (99.1 to 288) | 0,0001 |
| Physical activity_high | 3,7 | (0.4 to 7) | 0,0284 |
| Physical activity_moderate | 3,3 | (-0.3 to 6.8) | 0,0729 |
| Physical activity_low | Ref.^2^ |  |  |
| Age | -5,8 | (-10.5 to -1.2) | 0,0139 |
| Age*Age^3^ | 0,1 | (0 to 0.2) | 0,0082 |
| Age*Age*Age^4^ | 0,0 | (0 to 0) | 0,0046 |
| Sex_female | 0,9 | (-2 to 3.9) | 0,5283 |
| Sex_male | Ref. | (0 to 0) |  |
| Multimorbidity_no | -2,1 | (-5.6 to 1.4) | 0,2361 |
| Multimorbidity_yes | Ref. | (0 to 0) |  |
| EQVAS^5^ | 8,2 | (1.4 to 15) | 0,0183 |
| EQVAS*EQVAS^6^ | -2,2 | (-3.3 to -1.1) | 0,0001 |
| PHQ^7^ | 0,6 | (-0.2 to 1.5) | 0,1523 |
| PHQ*PHQ^8^ | -0,1 | (-0.1 to 0) | 0,0158 |
| BMI^9^ < 30 | 0,9 | (-2.3 to 4.1) | 0,5864 |
| BMI ≥ 30 | Ref. | (0 to 0) |  |
| Social network_cohabiting | 0,3 | (-3 to 3.5) | 0,8670 |
| Social network_solitarily | Ref. | (0 to 0) |  |
| Smoking_current | 3,0 | (-1.5 to 7.5) | 0,1899 |
| Smoking_former | 1,7 | (-1.4 to 4.9) | 0,2694 |
| Smoking_never | Ref. | (0 to 0) |  |
| Former stroke_no | 4,7 | (1.4 to 8) | 0,0048 |
| Former stroke_yes | Ref. | (0 to 0) |  |
| NIHSS^10^ | -0,7 | (-1.3 to -0.2) | 0,0131 |
| mRS^11^_2 | 0,0 | (-4.7 to 4.7) | 0,9996 |
| mRS_3 | -2,4 | (-6.8 to 1.9) | 0,2754 |
| mRS_4 | -6,9 | (-11.8 to -2) | 0,0054 |
| mRS_5 | -7,4 | (-12.8 to -2) | 0,0075 |
| mRS_6 | 2,7 | (-9.8 to 15.2) | 0,6705 |
| mRS_1 | Ref. |  |  |
| 1 Confidence Interval | 9 Body Mass Index, BMI = kg/m² | |  |
| 2 Reference Group | 10 National Institutes of Health Stroke Scale | | |
| 3 Age variable, squared | 11 European Quality of Life visual analogue scale | | |
| 4 Age variable, cubed |  |  |  |
| 5 European Quality of Life visual analogue scale (general health status) | | |  |
| 6 EQVAS variable, squared |  |  |  |
| 7 Patient Health Questionnaire (depressiveness) | |  |  |
| 8 PHQ variable, squared |  |  |  |
