# Supplementary material for: Associations between pre-stroke physical activity and physical quality of life three months after stroke in patients with mild disability
Source: PLoS One. 2022 Jun 29;17(6):e0266318. doi: 10.1371/journal.pone.0266318 (PMC9242505; doi:10.1371/journal.pone.0266318)
Supplement: S8 Table — (DOCX) [file pone.0266318.s011.docx]

| **S8 Table. Associations between pro-stroke physical activity and the four SIS subscales of physical quality of life after three months per quantile: Results of the quantile regression analysis** | | | | | | | |
| --- | --- | --- | --- | --- | --- | --- | --- |
| Stroke Impact Scale | Quantile | Moderate^1^ | | | High | | |
|  |  | Beta | (95 % CI) | p-value | Beta | (95 % CI) | p-value |
| Physical domain | 0,1 | 10,1 | (4.2 to 16) | 0,0009 | 5,0 | (-1 to 11) | 0,1030 |
|  | 0,2 | 4,7 | (0.3 to 9.1) | 0,0373 | 5,2 | (1.3 to 9.1) | 0,0088 |
|  | 0,3 | 3,5 | (-0.4 to 7.3) | 0,0775 | 5,8 | (3 to 8.6) | 0,0001 |
|  | 0,4 | 3,3 | (0.6 to 6.1) | 0,0183 | 3,4 | (1.5 to 5.4) | 0,0007 |
|  | 0,5 | 1,5 | (-0.7 to 3.7) | 0,1909 | 1,4 | (-0.3 to 3.1) | 0,1121 |
|  | 0,6 | 0,4 | (-1.7 to 2.5) | 0,6936 | 0,3 | (-1.4 to 2.1) | 0,7127 |
|  | 0,7 | 0,9 | (-0.7 to 2.6) | 0,2727 | 0,4 | (-1 to 1.8) | 0,5864 |
|  | 0,8 | 1,1 | (-0.6 to 2.8) | 0,1920 | -0,1 | (-1.5 to 1.3) | 0,8797 |
|  | 0,9 | 0,6 | (-0.8 to 2) | 0,4137 | -0,2 | (-1.5 to 1.1) | 0,7423 |
| Subdomain strenght | 0,1 | 2,2 | (-8.8 to 13.3) | 0,6913 | 2,9 | (-5.2 to 10.9) | 0,4840 |
|  | 0,2 | 2,8 | (-4.5 to 10.2) | 0,4503 | 5,2 | (0 to 10.5) | 0,0516 |
|  | 0,3 | 6,1 | (-0.5 to 12.6) | 0,0712 | 5,0 | (0.1 to 9.9) | 0,0439 |
|  | 0,4 | 3,8 | (-2.3 to 10) | 0,2211 | 3,3 | (-1.8 to 8.5) | 0,2073 |
|  | 0,5 | 3,1 | (-3.5 to 9.8) | 0,3516 | 3,7 | (-1.7 to 9.1) | 0,1769 |
|  | 0,6 | 4,1 | (-2.6 to 10.8) | 0,2327 | 3,0 | (-1.7 to 7.7) | 0,2052 |
|  | 0,7 | 5,1 | (-2.2 to 12.3) | 0,1691 | 2,7 | (-1.6 to 7.1) | 0,2178 |
|  | 0,8 | 4,7 | (-2.6 to 12) | 0,2032 | 2,1 | (-2.2 to 6.5) | 0,3347 |
|  | 0,9 | 4,9 | (-1.3 to 11.2) | 0,1207 | -1,9 | (-7.4 to 3.5) | 0,4906 |
| Subdomain hand function | 0,1 | 6,7 | (-5.3 to 18.7) | 0,2733 | 3,6 | (-6.7 to 14) | 0,4892 |
|  | 0,2 | 3,3 | (-4.9 to 11.6) | 0,4246 | 2,4 | (-4.4 to 9.1) | 0,4911 |
|  | 0,3 | 4,1 | (-1.2 to 9.4) | 0,1298 | 3,9 | (-0.3 to 8) | 0,0677 |
|  | 0,4 | 3,6 | (-0.7 to 7.8) | 0,0991 | 2,9 | (0 to 5.7) | 0,0464 |
|  | 0,5 | 1,6 | (-1.4 to 4.6) | 0,3048 | 0,2 | (-1.9 to 2.4) | 0,8204 |
|  | 0,6 | 2,0 | (0 to 4) | 0,0533 | -0,1 | (-2 to 1.8) | 0,8909 |
|  | 0,7 | 1,2 | (-0.3 to 2.7) | 0,1108 | -0,1 | (-1.4 to 1.1) | 0,8195 |
|  | 0,8 | 0,0 | (0 to 0) | 0,9873 | 0,0 | (0 to 0) | 0,9960 |
|  | 0,9 | 0,0 | (0 to 0) | 0,5044 | 0,0 | (0 to 0) | 0,8187 |
| Subdomain mobility | 0,1 | 11,0 | (3.4 to 18.7) | 0,0049 | 10,4 | (2.8 to 18.1) | 0,0077 |
|  | 0,2 | 1,6 | (-3.3 to 6.5) | 0,5293 | 3,2 | (-1.3 to 7.8) | 0,1650 |
|  | 0,3 | 1,7 | (-2.3 to 5.6) | 0,4112 | 2,6 | (-0.5 to 5.7) | 0,1014 |
|  | 0,4 | 1,7 | (-1.1 to 4.6) | 0,2279 | 1,2 | (-1 to 3.4) | 0,2715 |
|  | 0,5 | 0,6 | (-1.6 to 2.8) | 0,5774 | -0,1 | (-2 to 1.8) | 0,9096 |
|  | 0,6 | 0,8 | (-1.2 to 2.8) | 0,4406 | -0,6 | (-2.1 to 1) | 0,4763 |
|  | 0,7 | 0,3 | (-1.2 to 1.8) | 0,6807 | -0,6 | (-2 to 0.8) | 0,3835 |
|  | 0,8 | 0,7 | (-0.5 to 2) | 0,2418 | -0,3 | (-1.5 to 0.9) | 0,6429 |
|  | 0,9 | 0,3 | (-0.5 to 1.1) | 0,4630 | 0,0 | (-0.8 to 0.8) | 0,9710 |
| Subdomain activities of daily living | 0,1 | 8,0 | (1.6 to 14.5) | 0,0149 | 4,9 | (-2.6 to 12.3) | 0,1986 |
|  | 0,2 | 2,0 | (-2.7 to 6.8) | 0,3996 | 2,9 | (-2 to 7.8) | 0,2418 |
|  | 0,3 | 1,1 | (-2.6 to 4.8) | 0,5469 | 3,3 | (0.4 to 6.3) | 0,0271 |
|  | 0,4 | 2,1 | (-0.6 to 4.8) | 0,1305 | 2,0 | (0.3 to 3.8) | 0,0236 |
|  | 0,5 | 1,3 | (-0.6 to 3.2) | 0,1827 | 0,8 | (-0.6 to 2.3) | 0,2467 |
|  | 0,6 | 0,9 | (-0.5 to 2.3) | 0,1893 | -0,2 | (-1.3 to 1) | 0,7594 |
|  | 0,7 | 0,9 | (-0.3 to 2) | 0,1584 | -0,1 | (-1.1 to 1) | 0,8916 |
|  | 0,8 | 0,4 | (-0.6 to 1.4) | 0,4517 | 0,2 | (-1 to 1.3) | 0,7459 |
|  | 0,9 | 0,0 | (-0.6 to 0.7) | 0,9457 | 0,0 | (-0.6 to 0.7) | 0,9322 |
| Models adjusted for age, sex, multimorbidity, general health, mental health, weight status, social network, smoking, former stroke events, stroke severity (NIHSS, mRS) | | | | | |  |  |
| 1 Reference category for all variables: Low physical activity | | | |  |  |  |  |
|  |  |  |  |  |  |  |  |
