# Supplementary material for: Associations between pre-stroke physical activity and physical quality of life three months after stroke in patients with mild disability
Source: PLoS One. 2022 Jun 29;17(6):e0266318. doi: 10.1371/journal.pone.0266318 (PMC9242505; doi:10.1371/journal.pone.0266318)
Supplement: S1 Appendix — (PDF) [file pone.0266318.s012.pdf]

S1 Appendix: List of relevant diseases for defining multimorbidity

- Hypertension
- Circulatory disorders in the legs (Intermittent claudication, except varicoses)
- Coronary heart disease / Heart attack (Circulatory disturbances in the heart)
- Constrictions in the cervical vessels
- Chronic heart failure
- Disease of the heart valves
- Cardiomyopathy
- Atrial flutter / Atrial fibrillation
- Vasculitis
- Aneurysm in the brain vessels
- Persistent foramen ovale
- Obstructive sleep apnea syndrome (OSAS)
- Chronic bronchitis / Chronic obstructive pulmonary disease (COPD)
- Allergies
- Increased cholesterol levels in blood / Blood lipid elevation
- Osteoporosis
- Epilepsy
- Migraine
- Depression
- Anxiety disorder / Panic attack
- Malignant tumour / Cancer
- Diabetes mellitus
- Taken from free text statements
  - Allergic asthma
  - Aortic aneurysm
  - Asbestosis
  - Asthma
  - Bronchial asthma
  - Autoimmune hepatitis type 1
  - Abdominal aortic aneurysm
  - Chronic cor-pulmonale
  - Chronic renal insufficiency
  - Dementia
  - Diabetic nephropathy
  - Hepatitis type B
  - Liver cirrhosis
  - Pulmonary emphysema
  - Pulmonary fibrosis
  - Renal insufficiency
  - Renal insufficiency, dialyse
  - Renal insufficiency
  - Renal insufficiency stage 3
  - Kidney failure
  - Polyneuropathy
  - Polycystic kidney disease

- Pulmonary hypertension
- Pulmonary embolism
- Hepatitis
- Pulmonary embolism
- Kidney transplant
